# Effect of Bleomycin Hydrolase Expression in Tumor Tissue on the Therapeutic Effectiveness of Electrochemotherapy

**DOI:** 10.3390/cancers18010127

**Published:** 2025-12-30

**Authors:** Jan Bogataj, Ursa Lampreht Tratar, Gregor Sersa, Maja Cemazar, Ales Groselj, Masa Omerzel

**Affiliations:** 1 Department of Experimental Oncology, Institute of Oncology Ljubljana, 1000 Ljubljana, Slovenia; bogataj.jan@gmail.com (J.B.); ulampreht@onko-i.si (U.L.T.); gsersa@onko-i.si (G.S.); mcemazar@onko-i.si (M.C.); 2 Faculty of Medicine, University of Ljubljana, 1000 Ljubljana, Slovenia; 3 Veterinary Faculty, University of Ljubljana, 1000 Ljubljana, Slovenia; 4 Faculty of Health Sciences, University of Ljubljana, 1000 Ljubljana, Slovenia; 5 Faculty of Health Sciences, University of Primorska, 6310 Izola, Slovenia

**Keywords:** electrochemotherapy, bleomycin hydrolase, predictive biomarker, murine tumors

## Abstract

**Background:** Electrochemotherapy (ECT) enhances the intracellular delivery of chemotherapeutic agents, most notably bleomycin, through electroporation. Despite high response rates, tumor sensitivity to ECT varies, highlighting the need for predictive biomarkers. Bleomycin hydrolase (BLMH), an enzyme that metabolically inactivates bleomycin, may influence treatment outcomes. This study investigated the relationship between BLMH expression and bleomycin-based ECT effectiveness. **Methods:** BLMH expression was evaluated at the mRNA and protein levels in six murine tumor cell lines and their corresponding syngeneic tumors using qPCR, immunofluorescence, and immunohistochemistry. Correlations between BLMH expression and tumor response to ECT were assessed both in vitro and in vivo. **Results:** BLMH expression varied significantly among tumor models, without consistent patterns across cancer types. In vitro, BLMH mRNA levels strongly correlated with IC30 values for bleomycin (R = 0.74), while the correlation weakened at IC50 doses, suggesting enzyme saturation. In vivo, BLMH expression levels moderately correlated with complete tumor response rates following ECT (R = 0.50). Differences between in vitro and in vivo expression highlighted the role of the tumor microenvironment. **Conclusions:** High BLMH expression reduces tumor sensitivity to bleomycin-based ECT, supporting its role as a predictive biomarker. Measuring BLMH levels may help stratify patients and personalize ECT application; however, it is not the sole factor for response prediction. Future studies in clinical tumor samples are warranted to evaluate its predictive value and to develop integrated biomarker models.

## Simple Summary

Electrochemotherapy (ECT) is a cancer treatment that combines application of short electrical pulses with chemotherapy drugs to improve their delivery into tumor cells. Bleomycin is one of the most effective drugs used in ECT, but its success can vary depending on the tumor type. One possible reason is the presence of bleomycin hydrolase (BLMH), an enzyme that inactivates bleomycin. In this study, we measured BLMH levels in different mouse tumor cell lines and tumors grown in animals. Thereafter we compared the levels of BLMH with tumor response to ECT and found a correlation with BLMH content, i.e., tumors with higher BLMH content responded poorly. These findings suggest that levels of BLMH could serve as a predictive marker for ECT effectiveness. Testing for BLMH before treatment may help personalize therapy and identify patients most likely to benefit from ECT.

## 1. Introduction

Electrochemotherapy (ECT) is an established local ablative therapy for tumors that is used in over 180 centers throughout Europe. This treatment utilizes reversible electroporation, in which short electric pulses increase the permeability of cell membranes, facilitating the intracellular delivery of molecules that otherwise have limited access [1]. While electroporation is also used in gene electrotransfer, ECT specifically increases the effectiveness of chemotherapeutic agents such as bleomycin and cisplatin. Bleomycin in particular experiences a significant increase in cytotoxicity (up to 1000-fold) when combined with electroporation [2,3,4]. ECT has demonstrated effectiveness across various histological types of skin tumors, including melanoma metastases, basal cell carcinomas, and squamous cell carcinomas. Recent advances have extended its application to deep-seated tumors using percutaneous, endoscopic, and laparoscopic methods [5,6,7,8,9].

While ECT achieves objective response rates of 60–80%, response varies by tumor type and size, with larger tumors and melanomas generally having poorer outcomes [1]. Previous treatments such as radio- or chemotherapy and surgery can also reduce the effectiveness of the treatment. Currently, patient selection for ECT depends on clinical factors such as tumor size, histological type, and previous oncology treatments [10,11]. However, there is also a need for biological markers that predict the individual response of tumors to ECT. The identification of such predictive factors could significantly improve our understanding of the variability of tumor responses and allow for personalization of treatment.

ECT effectiveness is influenced by several factors, including intrinsic tumor cell characteristics, features of the tumor microenvironment, and clinical parameters. Tumor cells can exert different sensitivity to ECT or differ in tumor genomics and mutational load, viral infection or radio-, chemotherapy ortargeted drug resistance [12,13,14,15]. In parallel, the tumor microenvironment plays a crucial role, such as through vascularization and oxygenation, extracellular matrix composition (influencing tissue conductivity and electric field distribution), and the presence of immune infiltrate [16,17,18]. Clinical factors further contribute to treatment outcome, including tumor histotype, size, and previous treatments [19,20].

Some of the biological factors that influence ECT effectiveness have already been extensively investigated. Tumor perfusion and vascular density can directly affect bleomycin delivery and intratumoral distribution. Extracellular matrix (ECM) density can influence drug diffusion and electric field homogeneity, immune infiltration modulates the durability of tumor control following ECT-induced immunogenic cell death [20]. Not only biological factors, but also intrinsic biophysical and mechanical properties of tumor cells, such as membrane structure and resistance to physical stress, can influence responsiveness to electroporation-based therapies [21].

One potential mechanism influencing the effectiveness of ECT is the enzymatic inactivation of bleomycin by bleomycin hydrolase (BLMH). BLMH, a highly conserved cytoplasmic cysteine peptidase, metabolically inactivates bleomycin, a key component in many cancer chemotherapy regimens [22,23,24,25]. While the role of BLMH in other diseases is known, its impact on ECT outcomes remains unexplored. Once bleomycin is trapped inside the cells after electroporation, high BLMH activity may become the dominant factor restricting the amount of active drug available to induce DNA damage [26]. Given the variable BLMH content in different tissues and the lack of information on BLMH content in different tumor types, we hypothesize that BLMH activity reduces the effectiveness of ECT.

The aim of this study was to investigate the role of BLMH as a predictive factor for the effectiveness of ECT in different tumor cell lines and murine tumor models. Specifically, we aimed to evaluate BLMH levels in different tumor cell lines in vitro and evaluate BLMH levels in different murine tumor models in vivo. Ultimately, our aim was to determine whether BLMH expression correlates with response to bleomycin-based ECT. BLMH was selected as a potential predictive factor due to its direct modulation of the effective intracellular drug concentration and the feasibility of its measurement.

## 2. Materials and Methods

### 2.1. Cell Lines

Six different murine tumor cell lines were used for the experiments. Murine melanoma B16F10, colon carcinoma MC38 (Kerafast, Newark, CA, USA), and mammary carcinoma TS/A (American Type Culture Collection (ATCC), Manassas, VA, USA) were cultured in Advanced Dulbecco’s Modified Eagle Medium (DMEM; Gibco, Thermo Fisher Scientific, Waltham, MA, USA). Mammary carcinoma cells 4T1, fibrosarcoma WEHI 164, and colon carcinoma CT26 (ATCC) were cultured in Advanced RPMI-1640 medium (Giubco). All media was supplemented with 5% (*v*/*v*) fetal bovine serum (FBS; Gibco), 10 mL/L L-glutamine (GlutaMAX; Gibco), and 1% (*v*/*v*) penicillin–streptomycin (stock solution, 10,000 U/mL, Gibco). The cells were maintained in a 5% CO_2_ humidified incubator at 37 °C. The cells were routinely tested and confirmed to be free from mycoplasma infection, using the MycoAlertTM PLUS Mycoplasma Detection Kit (Lonza Group Ltd., Basel, Switzerland).

Cell lines and tumor types were selected based on relevancy to clinical practice. Melanoma, (liver metastases of) colorectal carcinoma, breast carcinoma, and sarcomas are tumors commonly treated with ECT [1]. Their reported in vitro/in vivo sensitivity to BLM differ significantly [27].

### 2.2. Animals and Tumors

Ten-week-old female C57Bl/6NCrl (Charles River, Lecco, Italy) and BALB/cOlaHsd mice from Envigo RMS Srl (San Pietro al Natisone, Udine, Italy) were used for the animal experiments. C57Bl mice were used for the B16F10 and MC38 tumor models and BALB mice were used for the TS/A, 4T1, CT26, and WEHI 164 tumor model experiments. For tumor formation, 100 µL of tumor cells in saline were injected into the flanks of the mice at a concentration of 3 × 10^5^ cells/mL. The mice were housed under specific pathogen-free conditions in a carousel mouse IVC rack system (Animal Care Systems Inc., Centennial, CO, USA) at a relative humidity of 55 ± 10%, a temperature of 20–24 °C and a 12 h light/dark cycle. Food and water were provided ad libitum. The experimental procedures were performed in compliance with the guidelines for animal experiments of the EU directive (2010/63/EU) and with permission from the Veterinary Administration of the Ministry of Agriculture, Forestry and Food of the Republic of Slovenia (permission no. U34401-3/2022/17). The experimental procedures followed the ARRIVE, OBSERVE, and PREPARE guidelines [28,29,30]. When tumors reached 50 mm^3^, mice were sacrificed and tumors were excised. This tumor size was chosen because ECT is performed at approximately 50 mm^3^, and BLMH content at the time of the treatment is the most relevant for treatment outcome. One half was snap frozen in liquid nitrogen for further PCR experiments, and one half was imbedded in paraffin for further immunohistological staining.

The effectiveness of ECT data (% of CR in vivo and IC50 values in vitro), used for correlation curves, were taken from our previous studies [14,18]. Briefly, (i) in vivo ECT was performed when tumors reached 50 mm^3^. Three minutes after i.v. 5 mg/kg BLM injection, ECT was performed with 8 square-wave pulses, 1300 V/cm, 100 µs, and 1 Hz, and tumor diameters (a × b × c × π/6) were measured 3 times per week. (ii) In vitro ECT was performed in 1 × 10^6^ cells mixed with bleomycin using the same pulses as those in vivo. Cell viability was assessed 72 h later using the Presto Blue assay [14,18].

### 2.3. RNA Isolation from Cells and Tumors

To assess expression of BLMH in mRNA levels in different cell lines, RNA was extracted from 10^6^ cells. We performed the experiment three times and, each time, isolate mRNA from 2 T25 cell culture flasks (Thermo Fischer Scientific) from the same cell line. Total RNA was extracted using the peqGOLD Total RNA Kit (VWR International, Radnor, PA, USA) according to the manufacturer’s instructions.

Ten to fifteen milligrams of frozen tumor samples were first mechanically homogenized in liquid nitrogen using a mortar and pestle. The powder was transferred to Eppendorf tube and TRIzol Reagent was used for lysis, followed by RNA isolation using the peqGOLD Total RNA Kit, according to the manufacturer’s protocol.

Concentrations and purity of RNA were determined spectrophotometrically using a Qubit 4 Fluorometer (Invitrogen, Thermo Fisher Scientific). A total of 1000 ng RNA was then reverse transcribed into cDNA with the SuperScript VILO cDNA Synthesis Kit (Thermo Fisher Scientific), following the manufacturer’s protocol. Reverse transcription was performed on a Primus 25 advanced^®^ thermocycler (VWR) under the following conditions, 25 °C for 10 min, 42 °C for 60 min, and 85 °C for 5 min, to terminate the reaction. The resulting undiluted cDNA was stored at −80 °C until further use.

### 2.4. Immunofluorescent Staining In Vitro

For immunofluorescence staining of BLMH, 10,000 cells per well were seeded in 12-well removable chamber slides (Ibidi, Gräfelfing, Germany) one day before the procedure. The next day, the cells were fixed with 4% paraformaldehyde in PBS (PFA; Alfa Aesar, MA, USA) for 15 min at 37 °C and washed twice with Hanks’ Balanced Salt Solution (HBSS, with calcium and magnesium, Gibco, Thermo Fisher Scientific). Cells were then exposed for 10 min to wheat germ agglutinin (WGA, Thermo Fisher Scientific, 5 µg/mL, diluted in HBSS = 1:1000 dilution), which labels cell membranes, and were washed again twice. Thereafter, the cells were blocked with 5% donkey serum containing 0.05% Tween-20 for 1 h at room temperature and incubated with rabbit polyclonal anti-BLMH antibody (Antibodies-Online, ABIN6127522; 1:50 dilution in 2% donkey serum/0.05% Tween-20) for 2 h. After two washing steps, the samples were incubated with donkey anti-mouse IgG H&L Alexa Fluor 488 secondary antibody (Abcam, ab150105; 1:500 dilution in 2% donkey serum supplemented with 22.5 mg/mL glycine) for 1 h in the dark. Cell nuclei were counterstained with 3 µg/mL Hoechst solution (Hoechst 33342, Trihydrochloride, Trihydrate, Thermo Fisher Scientific) for 10 min. Again, cells were washed twice with PBS, the silicon wells were removed, and the slides were mounted with a Prolong Gold Diamond Antifade Mount (Thermo Fisher Scientific). Imaging was performed with an LSM 800 confocal microscope (Carl Zeiss, Tokyo, Japan) with a 63x oil immersion objective. The collected images were then visualized in Imaris software (Bitplane, Zurich, Switzerland). The median fluorescence intensity of BLMH/cell was quantified with Imaris software (Bitplane).

### 2.5. Immunohistochemical Staining

Consecutive 2 μm thick sections were cut from each paraffin block of tumor samples. Immunohistochemical staining was performed using an anti-bleomycin hydrolase (BLMH) antibody (AA 1–300, antibodies-online GmbH, Aachen, Germany) at a dilution of 1:1000. As a negative control, sections were processed without the primary antibody. Antigen retrieval was carried out with sodium citrate buffer (10 mM sodium citrate, pH 6) for 30 min at 95 °C. Following retrieval, tissue sections were incubated overnight at 4 °C with the primary antibody. Detection was achieved using a peroxidase-conjugated streptavidin-biotin system (CTS008, Anti-goat HRP-DAB Cell & Tissue Staining Kit, R&D Systems; ab64261, rabbit-specific HRP/DAB detection IHC kit (ABC), Abcam), followed by hematoxylin counterstaining. Five images of viable tumor tissue were acquired at 40× magnification (numerical aperture 0.85) using a DP72 CCD camera (Olympus) connected to a BX-51 microscope (Olympus). Immunohistochemical analysis consisted of determining the percentage of positive staining in 5 images of each tumor slide by three independent observers. IHC quantification was performed by manual scoring. Specifically, the percentage of positively stained tumor area was assessed independently by three independent observers. The final value for each tumor represented the mean of the three assessments.

### 2.6. Statistical Analysis

The comparison of means of more than two groups was statistically evaluated by one-way analysis of variance (one-way ANOVA) followed by Dunnett’s or Tukey’s multiple comparisons test. A *p*-value of <0.05 was considered to indicate statistical significance. The values on the graphs are presented as the mean (AM) ± standard error of the mean (SE). All in vitro experiments were performed three times with two technical replicates. In vivo experiments were performed in six tumors for each tumor model. For statistical analysis and preparation of graphs, GraphPad Prism 10.4.1 (La Jolla, CA, USA) was used.

All correlation analyses presented in the manuscript were performed using the Pearson correlation test, as the data met assumptions of linearity and approximate normal distribution.

## 3. Results

### 3.1. In Vitro Expression of BLMH in Different Tumor Cell Lines

First, we determined in vitro expression of BLMH in two different tumor cell lines, B16F10 and TS/A, both on an mRNA and protein level, to confirm that there was a good correlation between RNA and protein levels of BLMH.

**Figure 1:**
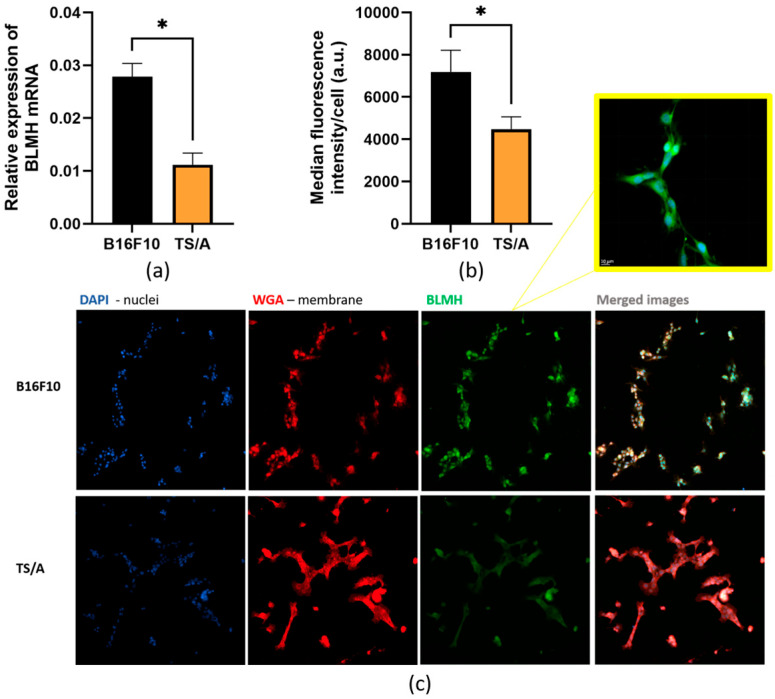
Expression of BLMH in B16F10 and TS/A cell lines. Expression of BLMH on mRNA (**a**) and protein level (**b**), with representative immunofluorescent images (**c**) showing BLMH protein in B16F10 and TS/A cells. Scale bar = 50 µm. For better visualization of cytoplasmic protein expression, the area is zoomed in on the yellow square. * *p* < 0.05 represents a statistically significant difference. Scale bar = 10 µm.

Relative expression of mRNA was significantly higher in the B16F10 cell line compared to in TS/A (Figure 1a). Cells were then stained for the presence of protein BLMH, which is localized in the cytoplasm (Figure 1b,c). Again, there was significantly higher expression of BLMH in B16F10 cells than in TS/A when fluorescent intensity of proteins was analyzed.

**Figure 2:**
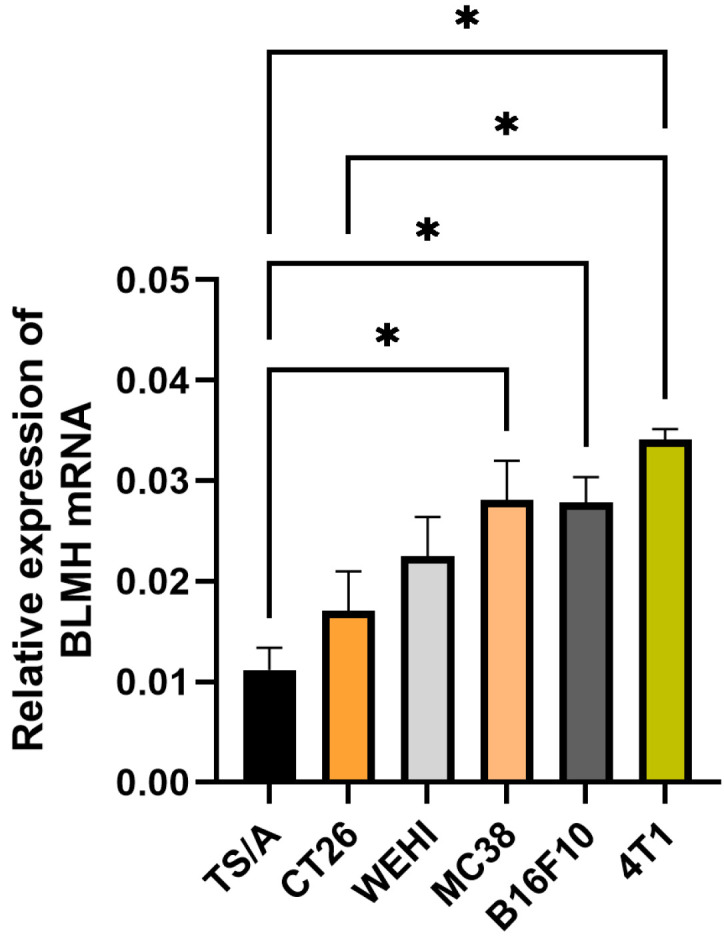
Expression of BLMH in six different tumor cell lines. * *p* < 0.05 represents a statistically significant difference.

Due to the good correlation between the mRNA and protein levels of BLMH in the cells, in other cell lines, only mRNA levels were measured (Figure 2). The expression pattern of BLMH varies among different tumor cell lines, as well as among cell lines originating from the same cancer type; i.e., the expression pattern of colon carcinoma cells (CT26 and MC38) or mammary carcinoma (TS/A and 4T1) was not similar. Moreover, there was a significant difference between the two mammary cell lines in BLMH mRNA expression (TS/A vs. 4T1, *p* < 0.05). However, the mentioned cell lines had different characteristics; 4T1 is a triple-negative breast cancer, whereas TS/A is an estrogen-positive and HER2-negative type [31]. These findings stress the importance of testing each individual cancer sample, since differences in cell characteristics and not only origin are important.

### 3.2. In Vivo Expression of BLMH in Different Murine Tumors

**Figure 3:**
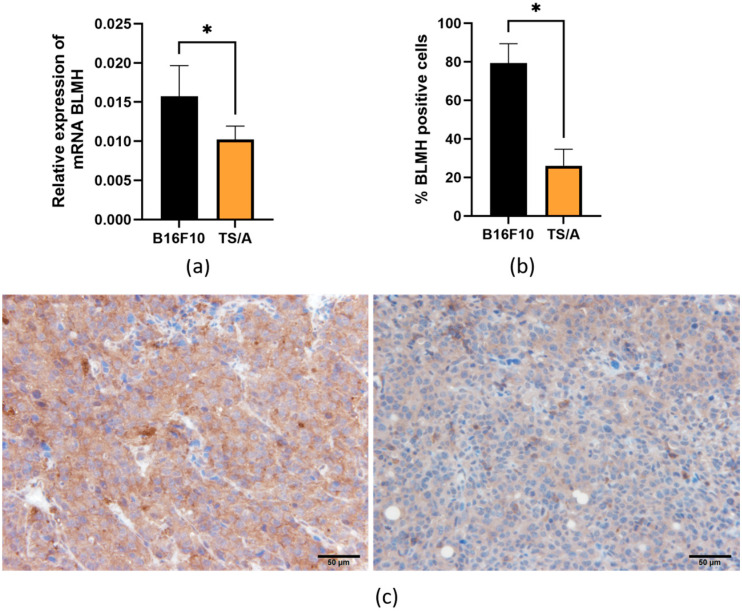
Expression of BLMH in two different tumor types. Expression on mRNA level (**a**) and protein level (**b**) with representative images of immunohistochemistry (**c**) showing B16F10 tumor (**left**) and TS/A (**right**). * *p* < 0.05 represents a statistically significant difference. Scale bar = 50 µm.

Expression of BLMH was then determined in six different murine tumors implanted from corresponding cell lines. Again, BLMH was first determined in two tumor models, B16F10 and TS/A, both on an mRNA (Figure 3a) and protein level (Figure 3b,c), to confirm that RNA levels of BLMH correlate relatively well with protein levels, also in vivo.

**Figure 4:**
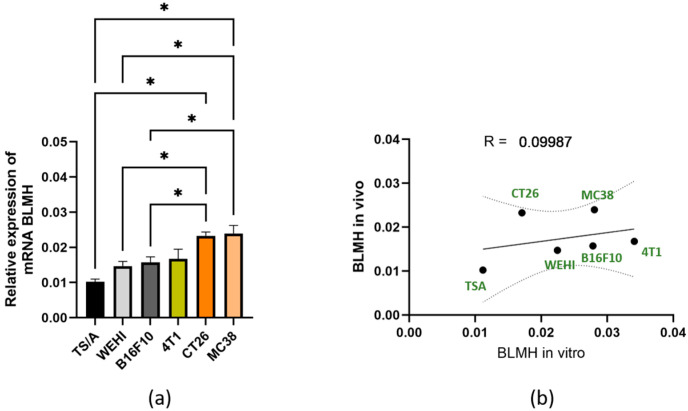
Expression of BLMH mRNA in vivo and correlation with in vitro expression. Expression of BLMH in six different tumors. * *p* < 0.05 represents a statistically significant difference. (**a**) Correlation test between in vitro and in vivo BLMH expression (**b**).

All other tumors were then tested for BLMH expression on an mRNA level (Figure 4a). As previously observed in cell lines, mRNA BLMH expression levels varied among different tumors with no specific pattern. No in vitro vs. in vivo correlation in mRNA expression levels was observed (Figure 4b).

### 3.3. Correlation Between mRNA Expression Levels and ECT Effectiveness

**Figure 5:**
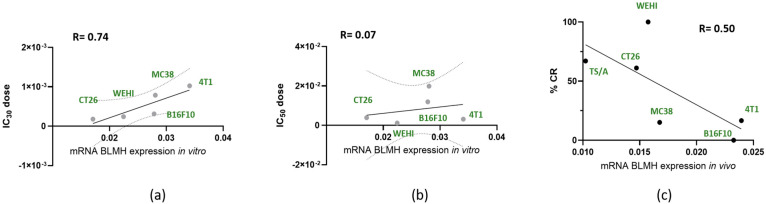
Correlation of mRNA BLMH expression with response. Expression of mRNA BLMH in vitro and correlation with IC_30_ (**a**) and IC_50_ doses (**b**). Expression of mRNA BLMH in vitro and correlation with tumor complete response (CR) percentage (**c**).

The correlation between the levels of BLMH and ECT effectiveness/cytotoxicity was first performed in vitro. The in vitro IC_30_ and IC_50_ doses, the doses causing death in 30% or 50% of cells, were taken from our previous published research [14,18]. Since not all in vitro values were in the same concentration range, Grubbs’ test was first performed to confirm that the most extreme value was a significant outlier from the rest (*p* < 0.05, two sided); therefore, the TS/A value was not used for further correlation calculations [32]. In addition to being a statistical outlier according to Grubbs’ test, TS/A cells display distinct biological characteristics, including lower aggressiveness and different bleomycin sensitivity compared to the remaining models. These biological differences likely contribute to the disproportionate IC values observed. Therefore, only five values were plotted (Figure 5a,b). We observed a strong correlation (R = 0.74) between mRNA BLMH expression and IC_30_ doses; however, with higher bleomycin doses, there was no correlation (R = 0.07), as shown in Figure 5b, where mRNA BLMH expression was correlated to IC_50_ doses.

The correlations were then tested in vivo. Again, values were tested with Grubbs’ test, but there were no significant outliers. We observed a moderate correlation (R = 0.50) between mRNA BLMH expression and percentage of tumor complete response after ECT (Figure 5c).

## 4. Discussion

ECT effectiveness depends on several different biological factors [20]. Some of them have been extensively studied and described in previous studies, while the current study focused on expression of BLMH in different tumor cell lines in vitro and tumor models in vivo [27]. A correlation between expression of BLMH and effectiveness of ECT was observed, although the strength of this correlation was moderate, showing that BLMH is not the sole factor predicting response to ECT.

BLMH is a cytoplasmic cysteine peptidase widely expressed in different tissue types. Its primary function is the metabolic inactivation of bleomycin, a chemotherapeutic glycopeptide antibiotic, through hydrolytic cleavage of the terminal amide group of its β-aminoalanine moiety. This enzymatic modification prevents bleomycin from binding to DNA and generating DNA damage, such as single- and, most importantly, double-strand breaks [22]. While BLMH is ubiquitously present in normal tissues, its expression levels vary considerably across cell types and organs, and it is also found in malignant tissues, where it may influence tumor sensitivity to bleomycin-based therapies. A study evaluating whether the single nucleotide polymorphism (SNP) A1450G in BLMH is linked to survival outcomes in testicular germ-cell cancer (TC) treated with bleomycin-based chemotherapy showed reduced survival and higher risk of early relapse for patients with the homozygous variant G/G genotype (compared to heterozygous A/G and wild-type A/A variant) [33]. In normal tissues that express little or no BLMH, particularly the skin and lungs, bleomycin can cause notable toxicity. Since these tissues have limited enzymatic capacity to inactivate bleomycin, they are more susceptible to drug-induced damage, leading to adverse effects such as skin hyperpigmentation, erythema, and pulmonary fibrosis [34,35,36,37].

The results of this study indicate that BLMH expression levels in tumor cells influence the therapeutic response to ECT [14]. We demonstrated that tumors with higher BLMH expression exhibited reduced sensitivity to bleomycin-based ECT, both in vitro and in vivo. Our data demonstrated a strong correlation between BLMH expression and bleomycin IC_30_ values in vitro (R = 0.74). It should be noted that the observed correlation is based on a limited number of cell lines (n = 5) and should therefore be interpreted as exploratory, underscoring the need for validation in larger datasets in future studies. In vivo, the linear correlation between BLMH expression and tumor complete response rate was moderate (R = 0.50), supporting the hypothesis that BLMH can serve as a partial biological predictive factor for tumor responsiveness to ECT; however, it is not the only predictive factor involved in the response. Also, the in vitro correlation weakened at higher bleomycin concentrations (IC_50_), suggesting that the cumulative dose of bleomycin in the cell is very high and that the enzyme could be saturated, therefore causing it to not efficiently metabolize the bleomycin. However, this assumption should be confirmed by future studies directly measuring BLMH enzymatic activity across increasing bleomycin concentrations.

We also observed a lack of correlation between in vitro and in vivo BLMH expression levels, which may be attributed to the complexity of the tumor microenvironment. Unlike isolated tumor cell lines, in vivo tumor tissue includes stromal, vascular, and immune components that contribute to drug distribution, metabolism, and immune-mediated effects [38]. The lack of in vitro and in vivo BLMH expression can contribute to tumor hypoxia, which induces metabolic and redox stress by HIF signaling, which can downregulate enzymes involved in drug metabolism [39,40]. This cannot be observed in 2D cell culture experiments. The other factor could be the presence of pro-inflammatory cytokines, which are present in the tumor microenvironment but absent in vitro. Pro-inflammatory cytokines such as TNF-α, IFN-γ, and IL-1β can suppress BLMH transcription through stress-response and NF-κB-dependent pathways. In addition, dense extracellular matrix and increased tissue stiffness in in vivo tumor models can alter integrin signaling and intracellular redox balance, influencing protease transcription and activity [41,42,43]. Moreover, notable differences in BLMH expression can be observed in different tumor subtypes (TS/A and 4T1) with the same tissue origin. Although TS/A and 4T1 are both mammary carcinomas and have a BALB/c background, they differ markedly in intrinsic tumor state, which can confound biomarker interpretation. 4T1 tumors are highly aggressive, metastatic, and immunologically “cold,” whereas TS/A tumors are less invasive and exhibit different stromal and immune interactions. These intrinsic differences likely influence BLMH regulation and underscore why histological origin alone is insufficient for biomarker prediction. This discrepancy highlights the need for careful evaluation of BLMH expression in clinically relevant tumor samples rather than relying solely on cell line models.

Differential BLMH expression among tumor types could explain variability in response to treatments such as ECT. Thus, BLMH expression levels hold potential as predictive biomarkers of treatment outcomes. In addition, while BLMH expression clearly contributes to treatment resistance, it is not the only factor reducing ECT effectiveness. Other biological factors, including drug uptake efficiency, efflux transporters, and DNA repair capacity as well as the tumor microenvironment, such as the tumor extracellular matrix, vasculature, or immune infiltrate, may also modulate responses to ECT. In a study, Groselj et al. investigated tumor vasculature and the effectiveness of ECT and demonstrated the importance of vascularization, which directly influences the pharmacokinetics of bleomycin such as through drug accumulation and intratumoral distribution [18]. Distribution of bleomycin in tumors could also be important for effective degradation with BLMH. In addition to bleomycin pharmacokinetics, the immune status of the tumor is also very important for the effectiveness of ECT. In general, immunologically “hot” tumors respond better to ECT alone compared to “cold” tumors [27]. ECT causes cell death (partially also immunogenic cell death) and consequently antigen release. In “hot” tumors containing pre-existing immune cell infiltration and inflammatory signaling, ECT can effectively amplify tumor-specific T-cell responses. In contrast, “cold” tumors lack immune activation, limiting immune-mediated effects of ECT and often requiring combined therapies [44].

Our data demonstrate a moderate in vivo correlation (R = 0.50) between tumor BLMH expression and CR rates, indicating BLMH is not the sole factor predicting tumor response. The interplay of several factors, such as tumor cell characteristics, tumor microenvironment characteristics (vascularization, ECM, and immune infiltrate), and clinical factors (tumor histotype, tumor size, and previous treatments) are crucial for ECT effectiveness. Therefore, future studies integrating BLMH assessment with other molecular and microenvironmental markers could help establish a composite predictive model for patient selection and treatment personalization.

One of the drawbacks of our study is that we determined only the expression and not the activity of BLMH, since enzymatic activity directly determines bleomycin inactivation. However, strong in vivo mRNA expression with response correlation indicates that expression itself is already a good predictive feature. Another drawback of this study is the lack of quantitative assessment of staining intensity and immunoreactivity scoring in the IHC samples. Inhibition of BLMH, either with siRNA or synthetic enzyme inhibitors, could further help to elucidate underlying mechanisms and presumably increase ECT effectiveness. Temporal profiling of BLMH following ECT could provide valuable insight into treatment-induced regulation. Good correlations between the mRNA and protein levels of BLMH in the cells was demonstrated in only two cell lines and may not be representative of all of them or future models.

From a translational perspective, the study suggests that BLMH could serve as one of the predictive biomarkers for the responsiveness of tumors to ECT. Measuring BLMH levels in tumor biopsies prior to treatment could potentially identify patients more likely to benefit from bleomycin-based ECT. Such stratification may be particularly important for tumors with variable or intrinsically high BLMH expression, where treatment outcomes are less favorable; however, clinical implementation would require standardized, validated assays, careful control of pre-analytical variables, and robust definition of clinically meaningful cut-off values to ensure reproducibility and accurate patient stratification. Nonetheless, further validation in clinical samples is essential to confirm the robustness and predictive value of BLMH in the heterogeneous setting of human cancers.

## 5. Conclusions

In summary, this study identifies a correlation between expression of BLMH and the effectiveness of ECT, although the strength of this correlation was moderate in vivo (and stronger in vitro), showing that BLMH is not the sole factor predicting the response. Its role as a predictive biomarker warrants further investigation in clinical cohorts, where it may contribute to improving therapeutic outcomes through precision application of electrochemotherapy. Further studies should validate BLMH expression at the protein and activity levels, as well as in human tumor biopsies. Another future direction could be to perform a multifactorial predictive model for patient stratification prior to ECT, including BLMH assessment with other biological and microenvironmental markers.

## Data Availability

All data needed to evaluate the conclusions are presented in the paper. All materials, data, and protocols described in the manuscript will be made available upon request.

## Abbreviations

The following abbreviations are used in this manuscript:

BLMH	Bleomycin hydrolase
CR	Complete response
ECT	Electrochemotherapy
